# Supplementary material for: Ethyl Gallate Isolated from Castanopsis cuspidata var. sieboldii Branches Inhibits Melanogenesis and Promotes Autophagy in B16F10 Cells
Source: Antioxidants (Basel). 2023 Jan 25;12(2):269. doi: 10.3390/antiox12020269 (PMC9952331; doi:10.3390/antiox12020269)
Supplement: Supplementary file 1 [file antioxidants-12-00269-s001.zip › antioxidants-2184836-supplementary.pdf]

Supplementary data

Ethyl Gallate Isolated from *Castanopsis cuspidata* var. *Sieboldii* Branches Inhibits Melanogenesis and Promotes Autophagy in B16F10 Cells

Moon-Hee Choi [1], Seung-Hwa Yang [2], Da-Song Kim [2], Nam-Doo Kim [3], and Hyun-Jae Shin [1,2,*]

Table captions

Table S1. Antioxidant effect results of CCSB EtOAc fraction 1-3.

Figure captions

Figure S1. (A) DPPH free radical scavenging activities and (B) ABTS cation radical scavenging activities of CCSB EtOAc fraction 1-3.

Figure S2. (A) ^{1}H-NMR spectrum of compound 1 in DMSO-d_6. (B) ^{13}C-NMR spectrum of compound 1 in DMSO-d_6, (C) LC-MS/MS profile of the ethyl gallate, (D) LC-MS/MS profile of the compound 1.

Figure S3. Measurement of (A) viability of B16F10 melanoma cells treated with CCSB and Measurement of melanin content with 5–20 μg/mL CCSB. Relative melanin content was determined at 72 h after treatment. n = 3, error bars, mean ± standard deviation. Effect of 100 μg/mL arbutin (367 μM) on melanin synthesis and tyrosinase activity in B16F10 cells. Significantly different compared with α-MSH, * $p < 0.05$, ** $p < 0.01$.α-MSH: α-melanocyte-stimulating hormone.

Figure S4. (A) Effect of the CCSB and arbutin on the tyrosinase (TYR), tyrosinase related protein 1 (TRP-1), and tyrosinase related protein 2 (TRP-2) protein expression in B16F10 melanoma cells. B16F10 melanoma cells were treated with the indicated concentrations of the CCSB and arbutin prior to α-melanocyte-stimulating hormone (α-MSH) treatment for 24 h. The loading control was assessed using a β-actin antibody. (B) Quantitative analysis of the TYR, TRP-1, TRP-2 by western blotting. Cell lysates were subjected to western blotting using antibodies against TYR, TRP-1, and TRP-2. * $p < 0.05$, ** $p < 0.01$, compared with α-MSH treatment.

Figure S5. (A) Effect of the CCSB and arbutin on the p-PKA, and p-CREB protein expression in B16F10 melanoma cells. B16F10 melanoma cells were treated with the indicated concentrations of the CCSB and arbutin prior to α-melanocyte-stimulating hormone (α-MSH) treatment for 24 h. The loading control was assessed using a β-actin antibody. (B) Quantitative analysis of the p-PKA, p-CREB by western blotting. Cell lysates were subjected to western blotting using antibodies against TYR, TRP-1, and TRP-2. * $p < 0.05$, ** $p < 0.01$, compared with α-MSH treatment.

Supplementary Table

Table S1.

Sample	DPPH IC_{50} (μg/mL)	ABTS IC_{50} (μg/mL)
Fr. 1	111.49 ± 1.60	111.74 ± 2.65
Fr. 2	88.50 ± 3.96	65.19 ± 1.72
Fr. 3	127.41 ± 0.48	126.79 ± 3.99

Supplementary Figures

Figure S1.

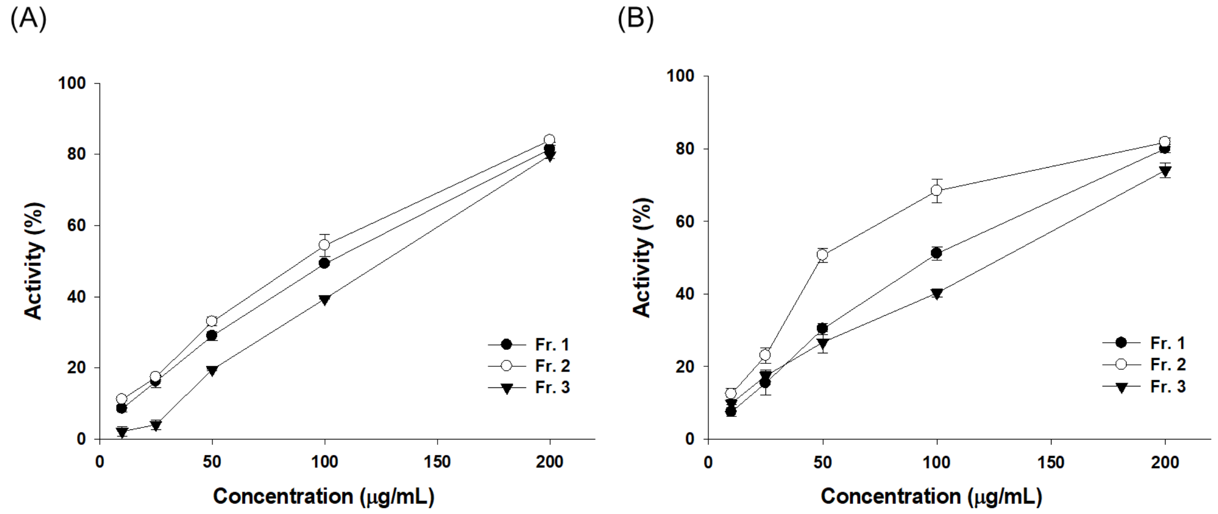

Figure S1. (A) DPPH free radical scavenging activities and (B) ABTS cation radical scavenging activities of CCSB EtOAc fraction 1-3.

Figure S2.

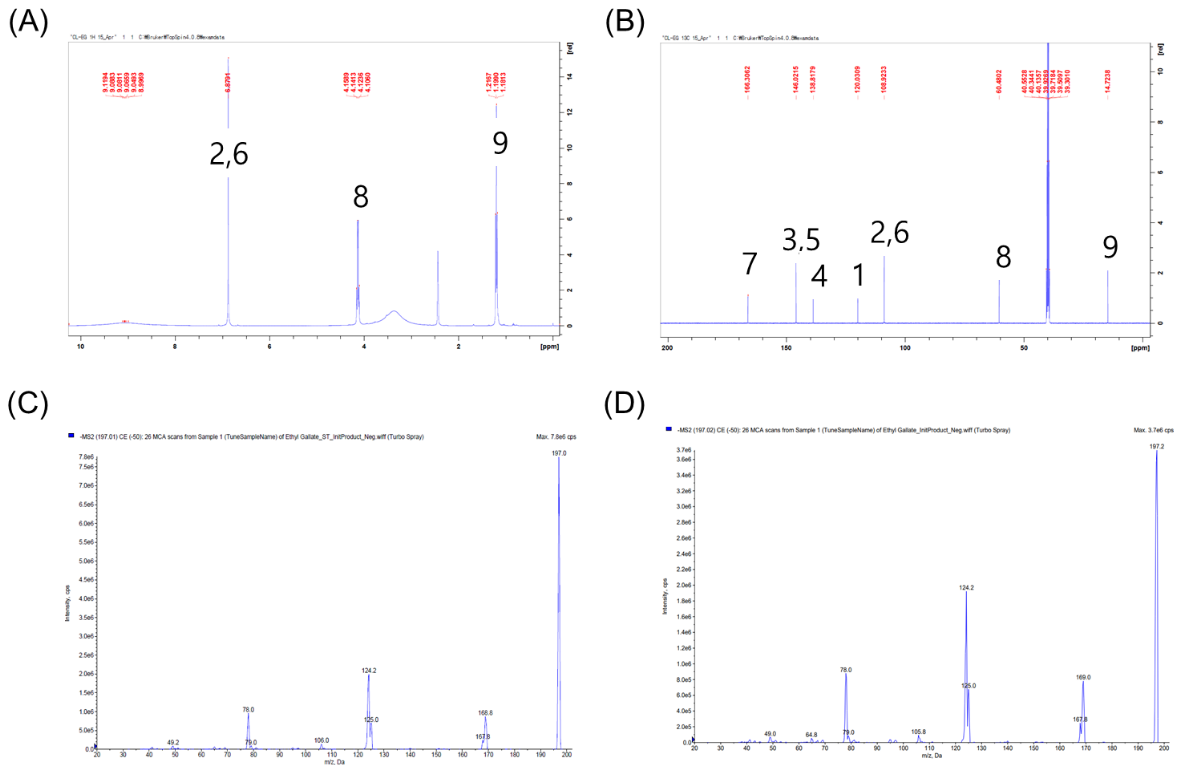

Figure S2. (A) ^{1}H-NMR spectrum of compound 1 in DMSO-d_6. (B) ^{13}C-NMR spectrum of compound 1 in DMSO-d_6, (C) LC-MS/MS profile of the ethyl gallate, (D) LC-MS/MS profile of the compound 1.

Figure S3.

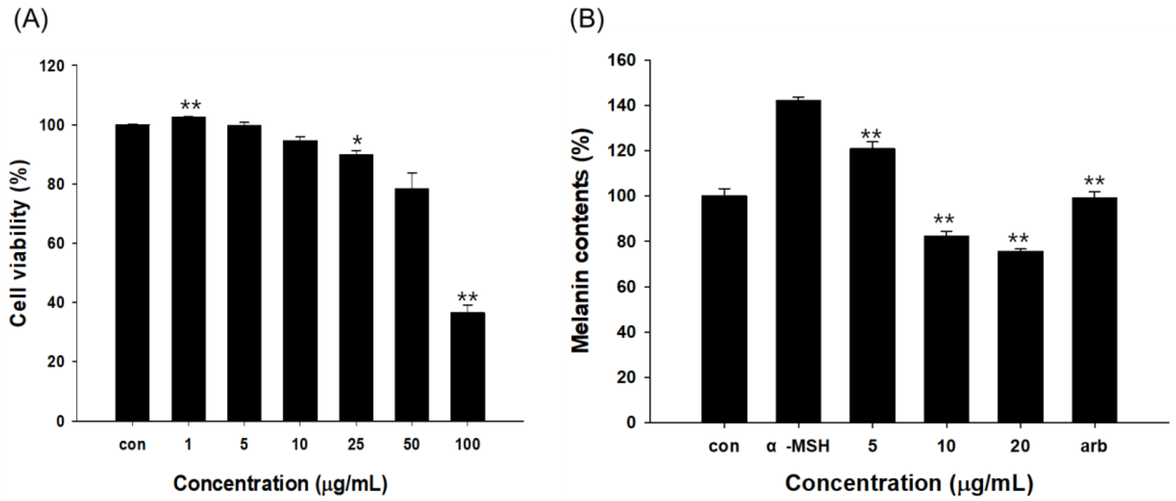

Figure S3. Measurement of (A) viability of B16F10 melanoma cells treated with CCSB and Measurement of (B) melanin content with 5–20 μg/mL CCSB. Relative melanin content was determined at 72 h after treatment. n = 3, error bars, mean ± standard deviation. Effect of 100 μg/mL arbutin (367 μM) on melanin synthesis and tyrosinase activity in B16F10 cells. Significantly different compared with α-MSH, * $p < 0.05$, ** $p < 0.01$.α-MSH: α-melanocyte-stimulating hormone.

Figure S4.

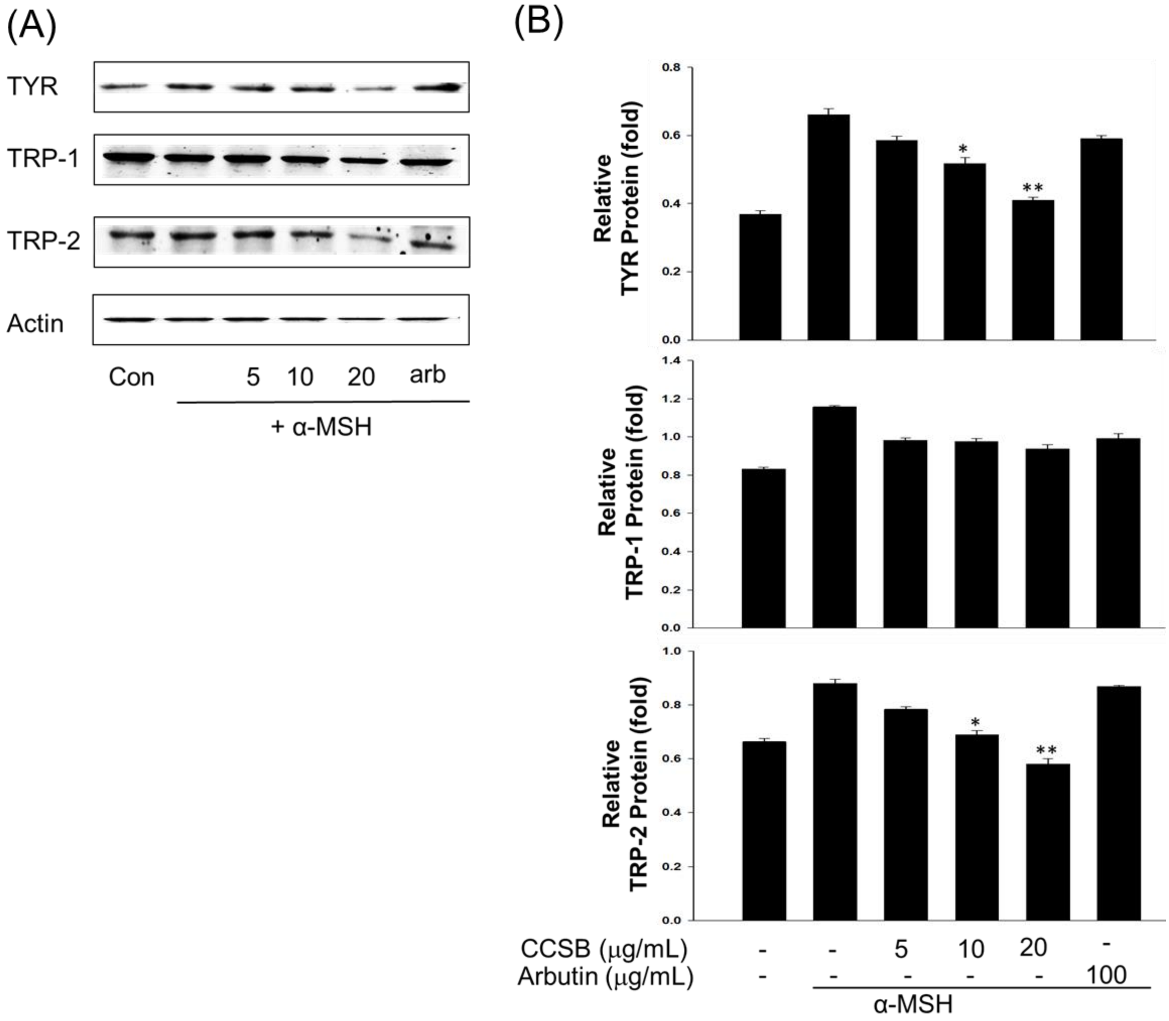

Figure S4. (A) Effect of the CCSB and arbutin on the tyrosinase (TYR), tyrosinase related protein 1 (TRP-1), and tyrosinase related protein 2 (TRP-2) protein expression in B16F10 melanoma cells. B16F10 melanoma cells were treated with the indicated concentrations of the CCSB and arbutin prior to α-melanocyte-stimulating hormone (α-MSH) treatment for 24 h. The loading control was assessed using a β-actin antibody. (B) Quantitative analysis of the TYR, TRP-1, TRP-2 by western blotting. Cell lysates were subjected to western blotting using antibodies against TYR, TRP-1, and TRP-2. * $p < 0.05$, ** $p < 0.01$, compared with α-MSH treatment.

Figure S5.

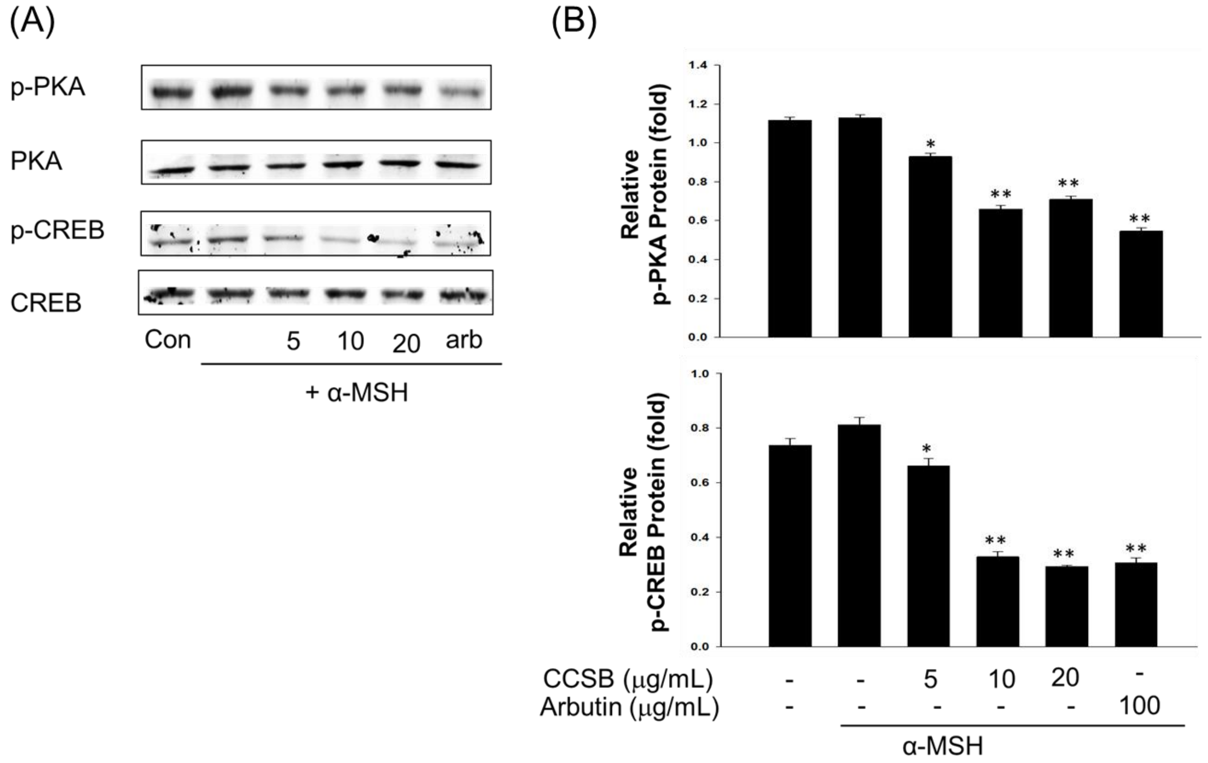

Figure S5. (A) Effect of the CCSB and arbutin on the p-PKA, and p-CREB protein expression in B16F10 melanoma cells. B16F10 melanoma cells were treated with the indicated concentrations of the CCSB and arbutin prior to α-melanocyte-stimulating hormone (α-MSH) treatment for 24 h. The loading control was assessed using a β-actin antibody. (B) Quantitative analysis of the p-PKA, p-CREB by western blotting. Cell lysates were subjected to western blotting using antibodies against TYR, TRP-1, and TRP-2. * $p < 0.05$, ** $p < 0.01$, compared with α-MSH treatment.
